# YY1 Is Required for Germinal Center B Cell Development

**DOI:** 10.1371/journal.pone.0155311

**Published:** 2016-05-11

**Authors:** Anupam Banerjee, Vishal Sindhava, Raja Vuyyuru, Vibha Jha, Suchita Hodewadekar, Tim Manser, Michael L. Atchison

**Affiliations:** 1 Department of Biomedical Sciences, University of Pennsylvania, School of Veterinary Medicine, 3800 Spruce Street, Philadelphia, Pennsylvania 19104, United States of America; 2 Department of Microbiology and Immunology, Thomas Jefferson University, 233 South Tenth Street, Philadelphia, Pennsylvania 19107, United States of America; COCHIN INSTITUTE, Institut National de la Santé et de la Recherche Médicale, FRANCE

## Abstract

YY1 has been implicated as a master regulator of germinal center B cell development as YY1 binding sites are frequently present in promoters of germinal center-expressed genes. YY1 is known to be important for other stages of B cell development including the pro-B and pre-B cells stages. To determine if YY1 plays a critical role in germinal center development, we evaluated YY1 expression during B cell development, and used a YY1 conditional knock-out approach for deletion of YY1 in germinal center B cells (CRE driven by the immunoglobulin heavy chain γ1 switch region promoter; γ1-CRE). We found that YY1 is most highly expressed in germinal center B cells and is increased 3 fold in splenic B cells activated by treatment with anti-IgM and anti-CD40. In addition, deletion of the *yy1* gene by action of γ1-CRE recombinase resulted in significant loss of GC cells in both un-immunized and immunized contexts with corresponding loss of serum IgG1. Our results show a crucial role for YY1 in the germinal center reaction.

## Introduction

Affinity maturation of immunoglobulins (Ig) in B cells largely occurs during the germinal center (GC) reaction where the processes of somatic hypermutation (SHM) and class switch recombination (CSR) occur [reviewed in references [1–3]]. B and T cells and that have been activated by antigen migrate to interfollicular regions in secondary lymphoid organs and interact [4,5]. These cells form long-lived interactions resulting in full B cell activation with increased expression of B Cell Lymphoma 6 (BCL6) protein and activation induced cytidine deaminase (AID) [6]. Activated cells migrate from the interfollicular region to the follicle where the B cells proliferate to begin formation of a germinal center [6,7]. Finally, the dark and light zones of the germinal center develop and B cells transition between these zones with SHM occurring in the dark zone, and affinity selection and CSR in the light zone. Ultimately the B cells that are selected, mature into either memory B cells or plasma cells and exit the germinal center [1,2].

A number of transcription factors regulate the germinal center reaction. BCL6 is critical for germinal center formation as its deletion ablates GC formation [6,8]. A variety of other transcription factors effect either early or late germinal center formation and include Pax5, IRF4, IRF8, NF-κB, E2A, c-Myc, MEF2B, MEF2C, EBF1, and SpiB [1–3]. In addition, the histone methyltransferase EZH2 is crucial for GC formation [9]. These factors regulate gene expression profiles needed for germinal center formation and control cell proliferation which approaches the highest rates in mammalian systems [10].

Recently, transcription factor Yin Yang 1 (YY1) was proposed to be a master regulator of germinal center function [11]. Using computational approaches, Green and colleagues [11] characterized promoters of genes that are expressed in germinal center cells. The promoters of these GC signature genes were enriched in binding sites for YY1. In addition, it has been proposed that YY1 binding sites, as well as sites for E2A and C/EBPα are enriched within non-immunoglobulin regions of the genome where AID binds and generates off-target site mutations, perhaps involved in genesis of B cell malignancies [12]. Consistent with this idea, we showed that YY1 physically interacts with AID, leading to its stabilization and nuclear accumulation [13]. We also found YY1 conditional knock-out in splenic B cells, results in reduction of CSR [13]. Furthermore, YY1 is known to be critical for B cell development at other B cell stages. Using mb1-CRE, the Shi laboratory showed that conditional deletion of the *yy1* gene in early pro-B cells results in pro-B cell arrest, reduced IgH locus contraction, and reduced VDJ rearrangement of distal Vh genes [14]. Similarly we showed that deletion of the YY1 REPO domain needed for recruitment of Polycomb Group (PcG) proteins to DNA results in arrest at the pre-B cell stage and highly skewed Vκ gene rearrangement patterns [15]. We also showed that YY1 physically interacts with, and co-localizes with proteins involved in long-distance DNA contacts including condensin, cohesin, and PcG subunits [15]. Thus, YY1 clearly plays a significant role in B cell development.

Here we evaluated YY1 expression during B cell development, and used a γ1-CRE conditional knock-out approach to delete YY1 in germinal center B cells. We found that YY1 is most highly expressed GC B cells. Deletion of the *yy1* gene resulted in significant loss of GC cells in both un-immunized and immunized contexts. Our results show a crucial role for YY1 in the germinal center reaction.

## Results

### YY1 is highly expressed in germinal center B cells

**Fig 1:**
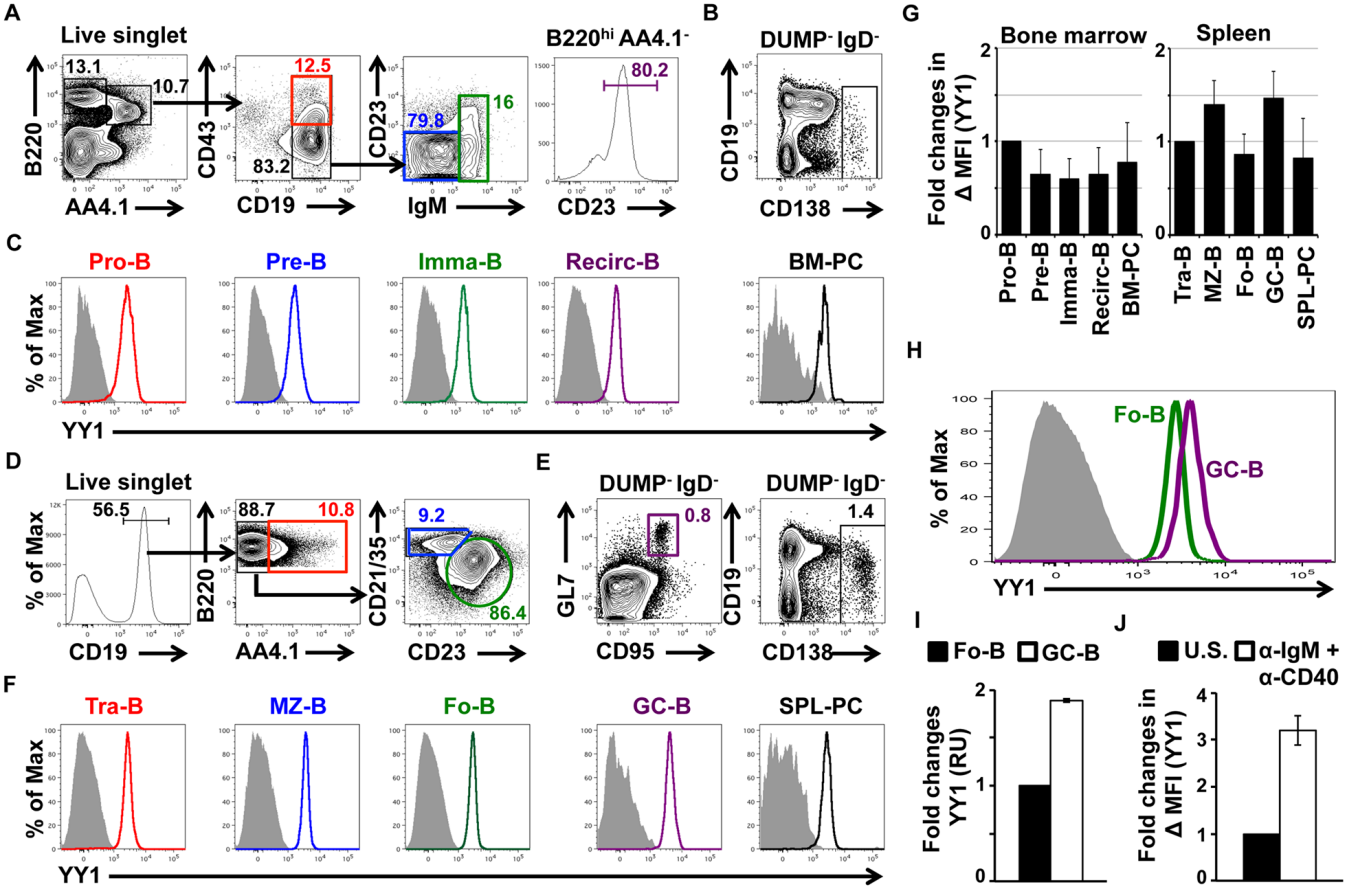
YY1 expression at various B cell developmental stages. (**A**) Bone marrow cells from non-immunized control Cγ1^Cre^ mice were stained with various antibodies to identify B cell developmental subsets, and **(B)** plasma cells. *A*. After doublet and dead cell discrimination, progenitor B cells (Pro-B) were phenotyped as B220^+^AA4.1^+^CD19^+^CD43^+^ cells; precursor B cells (Pre-B) were phenotyped as B220^+^AA4.1^+^CD19^+^CD43^-^CD23^-^IgM^-^ cells; immature B cells (Imma-B) were phenotyped as B220^+^AA4.1^+^CD19^+^CD43^-^CD23^+/-^IgM^+^ cells and recirculating mature B cells (Recirc-B) were phenotyped as B220^+^AA4.1^--^CD23^+^ cells. *B*. We used CD138 to detect bone marrow plasma cells (BM-PC). We gated on CD4^−^CD8^−^F4/80^−^Gr1^−^(DUMP gate)IgD^-^ cells that were CD138^+^. **(C)** Bone marrow B cell subsets and plasma cells (gated as shown in A & B) were stained with anti-YY1 antibody or corresponding isotype control. (**D**) Spleen cells from Cγ1Cre mice were stained with various antibodies to identify B cell subsets (it should be noted that our follicular B cell gating may contain a very small percentage of B1 cells), **(E)** germinal center B cells (GC-B) and plasma cells (SPL-PC). *D*. After doublet and dead cell discrimination, transitional B cells (Tra-B) were phenotyped as CD19^+^AA4.1^+^ cells; marginal zone B cells (MZ-B) were phenotyped as CD19^+^AA4.1^-^CD21/35^hi^CD23^lo^ cells and follicular B cells (Fo-B) were phenotyped as CD19^+^AA4.1^-^CD21/35^lo^CD23^hi^ cells. *E*. GL7 and CD95 were used to detect GC-B cells, and CD138 to detect SPL-PC. We gated on DUMP^-^IgD^-^ cells that were further subdivided into GL7^hi^CD95^hi^ GC-B and CD138^+^ SPL-PC. **(F)** Spleen B cell subsets, GC-B cells and SPL-PC (gated as shown in D & E) were stained with anti-YY1 antibody or corresponding isotype control. (**G)** Fold changes in mean fluorescence intensity (MFI) of YY1 in bone marrow B cell subsets (left panel, fold changes compared to pro-B cells) and spleen B cell subset (right panel, fold changes compared to tra-B cells). Fig A-G are representative results are from three independent experiments (*n* = 3 mice). **(H)** Comparison of YY1 staining of follicular and germinal center B cells. **(I)** Fo-B and GC-B cells were FACS sorted and YY1 expression was measured by qPCR. Expression was normalized to FO B cells (*n* > 3 mice). (J) Fold change in MFI of YY1 in un-stimulated (U.S.) or α-IgM + α-CD40 stimulated Fo-B cells after 3 days. Fig A-H are representative results from at least three independent experiments (*n* = 3 mice).

As YY1 is a ubiquitously expressed transcription factor that has been proposed to be important for germinal center B cell development, we set out to determine relative YY1 protein levels at each stage of B cell development. For this we performed FACS with a fluorescent antibody against YY1 on various B cell populations from non-immunized mice. In bone marrow we gated on cell surface markers for pro-B (B220^+^, AA4.1^+^, CD43^+^, CD19^+^), pre-B (B220^+^, AA4.1^+^, CD43^-^, CD19^+^, CD23^-^, IgM^-^), immature B (B220^+^, AA4.1^+^, CD43^-^, CD19^+^, IgM^+^), recirculating B (B220^+^, AA4.1^-^, CD43^-^, CD23^+^), and plasma cells (DUMP^-^, IgD^-^, CD138^+^) (see strategy in Fig 1A and 1B). Staining intensity with anti-YY1 for each B cell fraction is shown in Fig 1C. From spleen we gated on markers for marginal zone (B220^+^, AA4.1^-^, CD21/35^+^, CD19^+^, CD23^-^), follicular (B220^+^, AA4.1^-^, CD23^+^, CD19^+^, CD21/35^+^), germinal center (DUMP^-^, IgD^-^, GL7^+^, Fas^+^), and plasma B cells (DUMP^-^, IgD^-^, CDE138^+^) (see strategy in Fig 1D and 1E). Staining intensity with anti-YY1 for each B cell fraction is shown in Fig 1F. The comparative mean fluorescence intensity (MFI) in each B cell stage showed highest YY1 expression in germinal center B cells (Fig 1G and 1H).

By RT-PCR we found that YY1 transcripts were about 2 fold higher in germinal center B cells compared to follicular B cells (Fig 1I) corresponding with a roughly 2 fold increase in YY1 proteins levels as measured by MFI (Fig 1G and 1H). Activation of isolated splenic B cells with anti-IgM and anti-CD40 to mimic the germinal center activation phenotype caused a 3 fold increase in YY1 levels as measured by MFI (Fig 1J).

### YY1 deletion by γ1-CRE activity impacts splenic B germinal center populations

To determine the importance of YY1 in germinal center development, we used the *yy1*^*f/f*^ mouse line [14] that contains flox sites flanking the first exon in the *yy1* gene crossed with a γ*1CRE* line which expresses CRE recombinase from the IgH γ1 switch region promoter [16], to generate *yy1*^*f/f*^ γ*1CRE* mice. The γ*1CRE* gene is expressed upon B cell activation initiating the germinal center reaction and this results in deletion of floxed genes within the first two days of the germinal center reaction [1].

**Fig 2:**
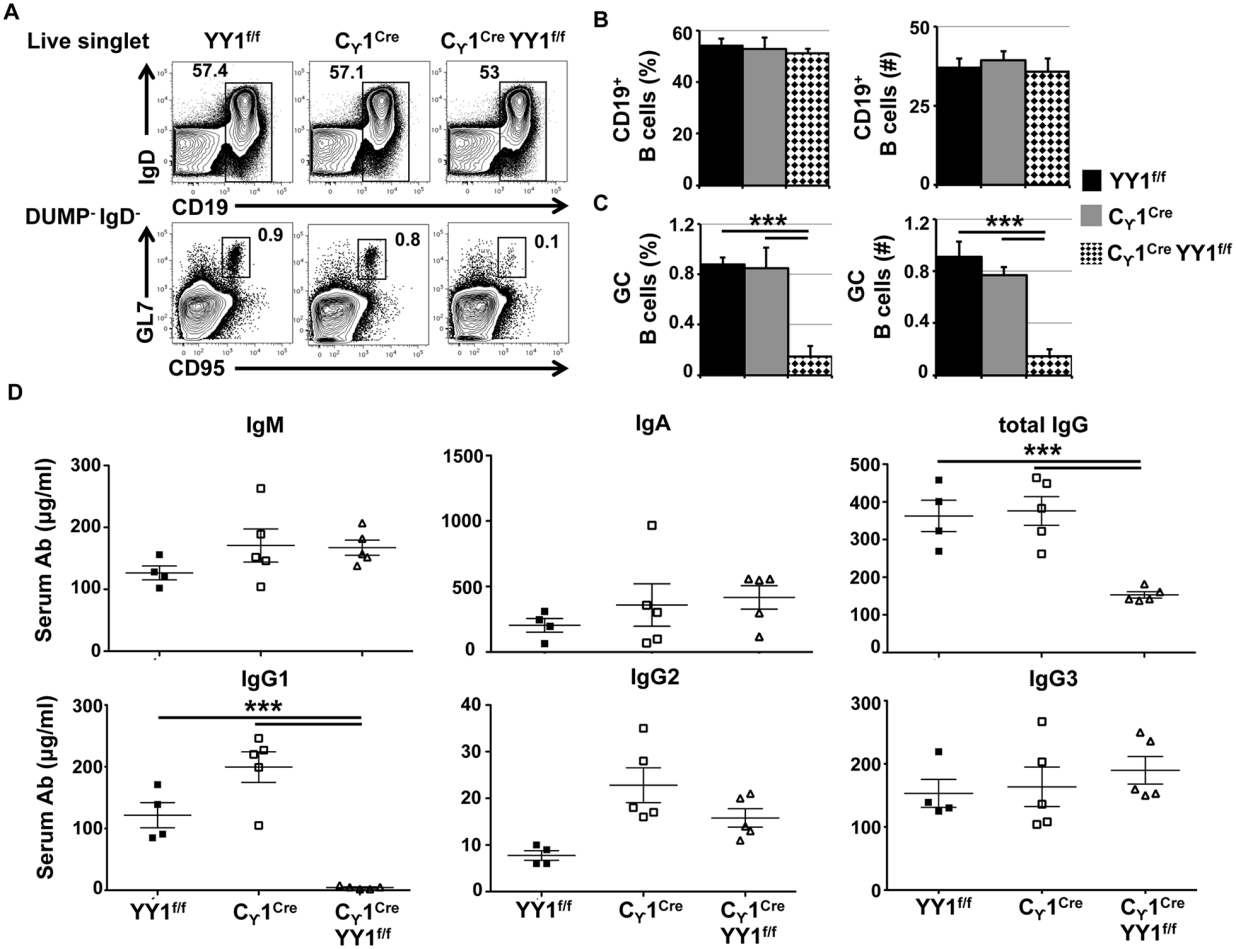
YY1 is required for germinal center B cell development and immunoglobulin class switching. **(A)** Spleen cells from non-immunized *YY1*^*f/*^, *γ1CRE* and *YY1*^*f/*f^ *γ1CRE* mice were stained with various antibodies to identify total B cells (CD19^+^AA4.1^+^, upper panel) and germinal center B cells (GC-B, DUMP^-^IgD^-^GL7^hi^CD95^hi^, lower panel). Percentages and number of **(B)** total B cells, and **(C)** GC-B cells per spleen of *YY1*^*f/*^, *γ1CRE* and *YY1*^*f/*f^ *γ1CRE* mice. Fig A-C are from three independent experiments (*n* = 3 mice for each genotype). **(D)** We used ELISA to detect various isotypes of serum immunoglobulins from *YY1*^*f/*^, *γ1CRE* and *YY1*^*f/*f^ *γ1CRE* mice. The concentration of IgM, IgA, total IgG, as well as IgG subclasses, IgG1, IgG2 and IgG3 were measured from sera samples that were obtained from four experiments (*n* ≥ 4 mice for each genotype). Asterisks indicate p<0.001.

Analyses of the naïve B cell populations present in spleen from non-immunized mice from each genotype (*yy1*^*f/f*^, γ*1-CRE*, and *yy1*^*f/f*^ *γ1-CRE*) showed that the transitional, marginal zone, follicular, and plasma B cell populations were relatively unchanged in each genotype (S1 Fig). Similarly, as expected, little differences were observed in bone marrow populations upon conditional deletion of YY1 by γ1CRE (S2 Fig). Thus, non-immunized *yy1*^*f/f*^, *γ1CRE*, and *yy1*^*f/f*^ *γ1CRE* mice showed the same levels of pro-B, pre-B, immature B, and recirculating B cells (S2 Fig). However, germinal center B cells showed a pronounced difference. Whereas *yy1*^*f/f*^ and *γ1CRE* mice showed germinal center B cell populations of 0.9–0.8% of total cells, this population dropped nearly 10 fold to 0.097% in *yy1*^*f/f*^ *γ1CRE* mice (Fig 2A). Whereas the percentage and number of total B cells remained unchanged, the percentage and number of germinal center B cells dropped dramatically in the *yy1*^*f/f*^ *γ1CRE* line compared to *yy1*^*f/f*^ and *γ1CRE* lines (Fig 2B and 2C). Thus, deletion of YY1 by action of the *γ1CRE* transgene resulted in loss in germinal center B cells. We observed no difference in the total number of T follicular helper (T_FH_) cells in the yy1^f/f^ *γ*1CRE mice compared to yy1^f/f^ and *γ*1CRE mice, which are essential for germinal center formation and maintenance (S3A and S3B Fig). In addition, we did not observe any difference in follicular B cell proliferation in response to various stimuli suggesting no adverse impact of CRE expression on follicular B cells in yy1^f/f^ *γ*1CRE mice (S3C Fig). Together, our results indicate that deletion of YY1 by action of the *γ*1CRE transgene resulted in loss of germinal center B cells.

We also measured serum Ig isotype levels in each genotype. As YY1 impacts germinal center B cell development where Ig CSR generally occurs, we anticipated that levels of IgM would remain similar, but that IgG1 isotype would be reduced in the *yy1*^*f/f*^ *γ1CRE* background due to activation of the γ1-CRE gene. As expected, we found that IgM levels were comparable amongst the unimmunized *yy1*^*f/f*^, γ*1CRE*, and *yy1*^*f/f*^ *γ1CRE* lineages as were IgA, IgG2, and IgG3 (Fig 2D). However, levels of IgG1, and total IgG were greatly reduced in the *yy1*^*f/f*^ *γ1CRE* line compared to the *yy1*^*f/f*^ and *γ1CRE* lines (Fig 2D).

### Antigen-specific GC cells are lost upon YY1 deletion

**Fig 3:**
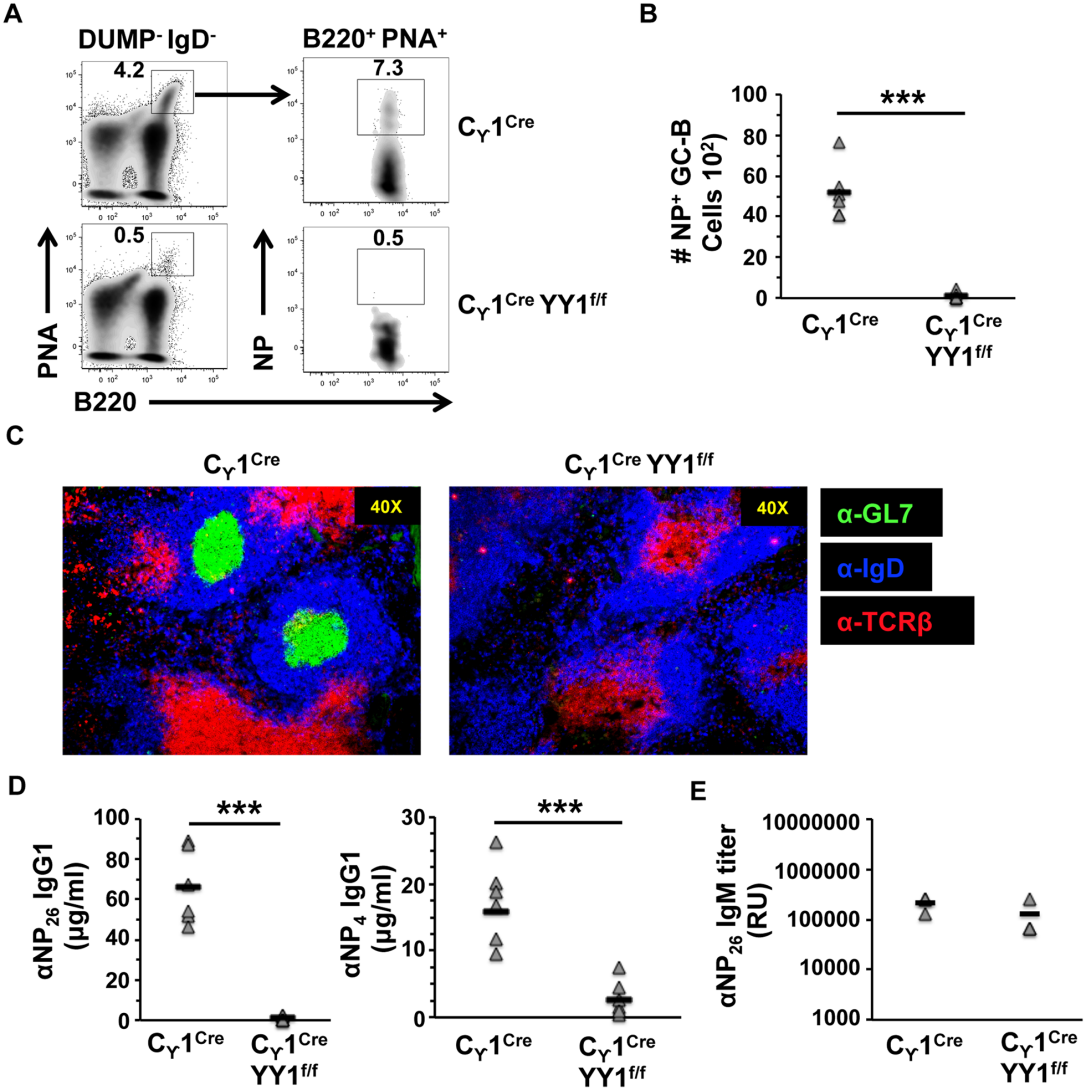
YY1 is required for antigen-specific germinal center development and for generation of antigen-specific IgG1. **(A)** Splenocytes from NP-CGG immunized *γ1CRE* and *YY1*^*f/*f^ *γ1CRE* mice were harvested at 14 days after immunization and stained with various antibodies, as well as PNA to detect GC B cells. We gated on CD4^−^CD8^−^F4/80^−^Gr1^−^(DUMP^−^) IgD^-^ cells that were subdivided into PNA^+^B220^+^ GC-B cells. GC-B cells were gated and further subsetted into NP-specific (NP^+^B220^+^) GC-B cells. Representative results are from three independent experiments. **(B)** Numbers of NP-specific (NP^+^B220^+^) GC-B cells per spleen of immunized mice (*n* = 3). **(C)** *γ1CRE* and *YY1*^*f/*f^ *γ1CRE* mice were immunized with NP-CGG, and 14 days later spleen sections were stained with anti-GL7, anti-IgD and anti-TCRβ antibody. GL7-rich regions demarcate germinal center B cells. **(D, E)** Serum from NP-CGG immunized *γ1CRE* and *YY1*^*f/*f^ *γ1CRE* mice were collected at 14 days after immunization and NP-specific serum Igs were analyzed using ELISA. *D*. The concentration of low affinity (NP26, left panel) and high affinity (NP4, right panel) IgG1 in the serum. *E*. Titer of NP-specific total IgM in the sera of immunized mice. Data are derived from sera samples that were obtained from three experiments. Asterisks indicate p<0.001.

To determine the impact of YY1 conditional knock-out on germinal center B cells after initiation of an immune response, we injected mice with NP-chicken gamma globulin (NPP-CGG), a T cell-dependent antigen. After two weeks we collected blood and spleen to determine immune responses. Deletion of YY1 by γ1-CRE action in the *yy1*^*f/f*^ *γ1CRE* line caused tremendous loss in the number of NP+ germinal center B cells compared to the *γ1CRE* line (Fig 3A and 3B). This was confirmed by staining histological sections with fluorescent antibodies that recognize germinal center B cells (anti-GL7), follicular B cells (anti-IgD), and T cells (anti-TCRβ) (Fig 3C). Thus, deletion of YY1 by γ1CRE caused loss of germinal center B cells, but not follicular B cells or T cells (Fig 3B).

We also determined the impact of YY1 deletion on high affinity verses low affinity antibodies against NP-CGG using NP(CGG)4 reactivity as definition of high affinity and NP(CGG)26 as low affinity. Deletion of YY1 caused a drop in both high affinity and low affinity IgG1 antibodies against NP-CGG (Fig 3D). In contrast, IgM antibodies against NP-CGG produced by cells that have not entered the germinal center reaction were unaffected (Fig 3E). These results demonstrate that YY1 is critical for germinal center B cell development, germinal center-mediated immune responses, and loss of YY1 ablates the formation of germinal centers.

## Discussion

Our results indicate that deletion of the *yy1* gene by action of γ1-driven CRE dramatically reduces the number of germinal center B cells in the spleen, as well as the histological appearance of germinal centers. The γ1 promoter is activated early in the germinal center reaction causing gene deletion within the first two days after antigen stimulation. This indicates that YY1 is critical for early events in germinal center development.

The phenotype with γ1CRE-mediated YY1 deletion is very similar to that observed upon EZH2 deletion using the same γ1CRE transgene [9]. EZH2 is a Polycomb Group (PcG) protein component of the Polycomb Repressive Complex 2 (PRC2) and is responsible for trimethylation of histone H3 on lysine 27 leading to stable transcriptional repression [17–22]. EZH2 is also involved in cell proliferation and germinal center B cells are among the highest proliferating cells in mammalian systems [10]. EZH2 expression increases in germinal center B cells indicating its apparent importance in transcriptional repression or proliferation [23,24]. EZH2 is implicated in a number of malignancies and importantly, is directly involved in development of various lymphomas including diffuse large B cell lymphoma (DLBCL) derived from germinal center B cells [9,25,26].

Previously we showed that YY1 can function as a PcG protein to mediate PcG-dependent transcriptional repression [27]. Importantly, we found YY1 can recruit PcG proteins to specific DNA sequences to control histone H3 lysine 27 tri-methylation [28–31]. Interestingly, we previously showed that YY1 physically interacts with EZH2 [15], and can control its ability to bind to specific DNA sequences in in the genome [27–32]. However, it is unclear whether YY1 controls EZH2 DNA binding in germinal center B cells.

YY1 now joins a growing list of transcription factors involved in germinal center development including BCL6, IRF4, IRF8, NF-κB, E2A, c-Myc, MEF2B, MEF2C, EBF1, and SpiB [1–3]. Each factor appears to control various aspects of germinal center biology by either regulating germinal center-specific gene regulatory networks, or by controlling proliferation. Deletion of the *c-myc* gene by the same γ1-CRE gene used here also results in loss of germinal center B cells. c-Myc is needed for early germinal center formation as well as for germinal center maintenance [33,34]. It is believed that the importance of c-myc for germinal center formation and maintenance relates to its ability to control cell proliferation. Germinal center B cells proliferate at an extremely high rate and loss of this proliferation likely relates to loss of germinal center initiation as well as collapse of germinal center maintenance.

YY1 was proposed to regulate germinal center biology by regulating gene expression networks, as YY1 DNA binding sites lie within the promoters of genes expressed in germinal center B cells [11]. Consistent with this hypothesis, we found that YY1 protein is expressed at highest levels in germinal center B cells. However, YY1 controls multiple stages of B cell development. For instance, YY1 deletion early in B cell development by action of mb1-driven CRE results in arrest at the pro-B cell stage and loss of immunoglobulin heavy chain (IgH) locus contraction needed for distal Vh gene rearrangement [14]. In addition, the long distance DNA contacts needed for V(D)J rearrangement at the pro-B cell stage are ablated upon YY1 deletion [35,36]. At the pre-B cell stage, we showed that YY1 PcG function is required for generating complete Igκ gene repertoires, again likely due to impacting long-distance DNA loops needed for Vκ-Jκ rearrangement [15]. Finally, we showed that YY1 is important in mature splenic B cells for controlling IgH class switch recombination (CSR) [13]. While germinal center-specific genes may be regulated by YY1, the requirement for YY1 early in the germinal center reaction, and the ability of YY1 to control other stages of B cell development suggest that YY1 either impacts numerous distinct B cell stage-specific functions, or it controls common functions at each stage such as cell survival or proliferation. Indeed YY1 controls numerous housekeeping genes such as the ribosomal protein genes among others [37–39], and its complete knock-out results in early embryonic lethality [40]. Therefore, additional work will be necessary to determine the multiple and varied roles of YY1 in B cell development.

## Materials and Methods

### Mice and Immunization

We obtained *yy1*^*f/f*^ mice from Dr. Yang Shi (Harvard University) and bred these mice with γ1-CRE mice generated by the Rawjewsky laboratory [16] and supplied by Jackson Laboratories (Stock No: 010611). Work using mice followed recommendations in the Guide for the Care and Use of Laboratory Animals of the National Institutes of Health. The protocol was approved by the Institutional Animal Care and Use Committee of the University of Pennsylvania (Protocol 803080). Mice were immunized i.p. with 50μg NP-CGG in alum.

### Flow Cytometry

For all analyses, bone marrow (BM) and spleens were disrupted to single cell suspension, and red blood cells were lysed using ACK buffer (Lonza). Equal numbers of cells were incubated with live/dead fixable aqua stain in PBS for 20 min at room temperature. Cells were washed and stained for surface antigens in PBS with 2% bovine serum albumin (BSA) for 30 min at 4°C. Following washing, cells were treated with Cytofix/Cytoperm buffer (eBiosciences) and then stained with antibodies against intracellular Ags for 30 min at 4°C. Data were collected on a BD LSR II flow cytometer and analyzed with FlowJo software (Tree Star). The antibodies used for flow cytometry were CD19 (clone 6D5), B220 (clone RA3-6B2), CD43 (clone S7), AA4.1/CD93 (clone AA4.1), CD23 (clone B3B4), CD21/35 (cloneBio4E3), IgD (clone 11–26), IgM (clone ll/41), CD4 (clone H129.19), CD8 (clone 53–6.7), F4/80 (clone BM8), Gr-1 (clone RB6-8C5), CD138 (clone 281–2), CD95/FAS (clone J02), GL7 (clone GL-7), CD62L (clone MEL-14), CXCR5 (clone RF8B2), FoxP3 (clone MF-14), PD-1 (clone J43), and YY1 (clone EPR4652). Exclusion of TOPRO-3 (Invitrogen) was used to identify live cells and doublets were excluded by forward and side scatter height versus width analysis.

### ELISA

Ninety-six well plates were coated with 10μg/ml anti-Ig (H + L) (Southern Biotech) overnight at 4°C and blocked with PBS containing 2% BSA for one hour. Sera were incubated at various dilutions for one hour at room temperature. Detection was conducted using HRP-conjugated goat anti-mouse IgM, IgG, IgG1, IgG2_a+c_, IgG3 or IgA (Southern Biotechnology) with a TMB substrate kit (BD Biosciences) and color development was quantified using EMax (Molecular Devices).

### Immunohistochemistry

Spleens from NP-CGG immunized mice were collected on day 14 and immersed in O.C.T. (Tissue Tek), flash frozen using 2-methylbutane cooled with liquid nitrogen, and stored at −20°C. 8–10 μm sections were sliced in a cryostat, fixed with cold acetone, rehydrated in PBS, and incubated with antibodies in PBS containing 10% serum. Sections were stained with anti-GL7, anti-IgD and anti-TCRβ antibodies.

## Supporting Information

S1 Fig Flow scheme of B cell subsets and plasma cells in the spleen. **(A)** Spleen cells from *yy1*^*f/f*^, *γ1CRE*, and *yy1*^*f/f*^ *γ1CRE* mice were stained with various antibodies to identify B cell subsets, and **(B)** plasma cells (SPL-PC). *A*. After doublet and dead cell discrimination, transitional B cells (Tra-B) were phenotyped as CD19^+^ B220^+^AA4.1^+^ cells; marginal zone B cells (MZ-B) were phenotyped as CD19^+^ B220^+^AA4.1^-^CD21/35^hi^CD23^lo^ cells and follicular B cells (Fo-B) were phenotyped as CD19^+^ B220^+^AA4.1^-^CD21/35^lo^CD23^hi^ cells. *B*. CD138 staining was used to detect SPL-PC. Splenocytes were gated on DUMP^-^IgD^-^ cells that were further subdivided into CD138^+^ SPL-PC.
(TIF)

S2 Fig Flow scheme of B cell subsets in the bone marrow. **(A)** Bone marrow cells from *yy1*^*f/f*^, *γ1CRE*, and *yy1*^*f/f*^ *γ1CRE* mice were stained with various antibodies to identify B cell developmental subsets, and re-circulating mature B cells. After doublet and dead cell discrimination, progenitor B cells (Pro-B) were phenotyped as B220^+^AA4.1^+^CD19^+^CD43^+^ cells; precursor B cells (Pre-B) were phenotyped as B220^+^AA4.1^+^CD19^+^CD43^-^CD23^-^IgM^-^ cells; immature B cells (Imma-B) were phenotyped as B220^+^AA4.1^+^CD19^+^CD43^-^CD23^+/-^IgM^+^ cells and recirculating mature B cells (Recirc-B) were phenotyped as B220^+^AA4.1^-^CD23^+^ cells.
(TIF)

S3 Fig Comparable T_FH_ cell numbers and Fo B cell proliferation in Cγ1Cre YY1^f/f^ mice. **(A)** Representative staining of T_FH_ cells in YY1^f/f^ mice. Live singlet cells were gated and subsetted into T_FH_ (CD4^+^FoxP3^−^CD62L^-^PD1^hi^CXCR5^hi^) cells. **(B)** Number of T_FH_ cells per spleen of non-immunized YY1^f/f^, Cγ1Cre and Cγ1Cre YY1^f/f^ mice. Representative gating strategy to identify T_FH_ (CD4^+^FoxP3^−^CD62L^-^PD1^hi^CXCR5^hi^) cells as shown in A (left). **(C)** MACS-sorted CD23^+^ Follicular B cells from YY1^f/f^, Cγ1Cre and Cγ1Cre YY1^f/f^ mice were labeled with CFSE and stimulated for 60 hours with anti-IgM (20 μg/ml), anti-IgM + anti-CD40 (2.5 μg/ml), LPS (5 μg/ml), or CpG (1 μM). At the end of the culture, live and dead cells were identified by TO-PRO-3 staining. CFSE dilution in the live cells is shown in the figure. Representative results are from three independent experiments.
(TIF)
