# Supplementary figures and images for: YY1 Is Required for Germinal Center B Cell Development
Source: PLoS One. 2016 May 11;11(5):e0155311. doi: 10.1371/journal.pone.0155311 (PMC4863967; doi:10.1371/journal.pone.0155311)

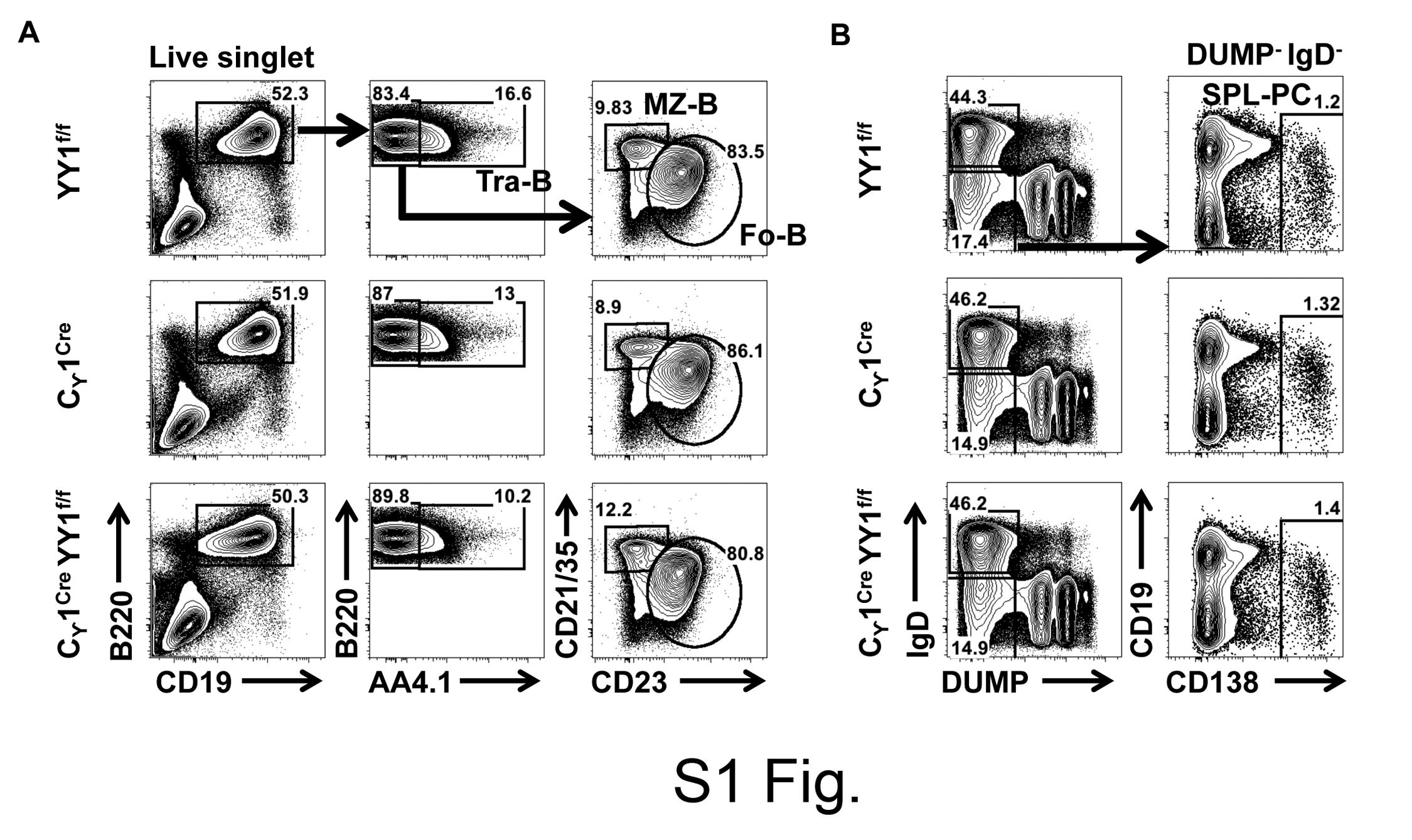

Supplement: S1 Fig — (A) Spleen cells from yy1f/f, γ1CRE, and yy1f/f γ1CRE mice were stained with various antibodies to identify B cell subsets, and (B) plasma cells (SPL-PC). A. After doublet and dead cell discrimination, transitional B cells (Tra-B) were phenotyped as CD19+ B220+AA4.1+ cells; marginal zone B cells (MZ-B) were phenotyped as CD19+ B220+AA4.1-CD21/35hiCD23lo cells and follicular B cells (Fo-B) were phenotyped as CD19+ B220+AA4.1-CD21/35loCD23hi cells. B. CD138 staining was used to detect SPL-PC. Splenocytes were gated on DUMP-IgD- cells that were further subdivided into CD138+ SPL-PC. (TIF) [file pone.0155311.s001.tif]

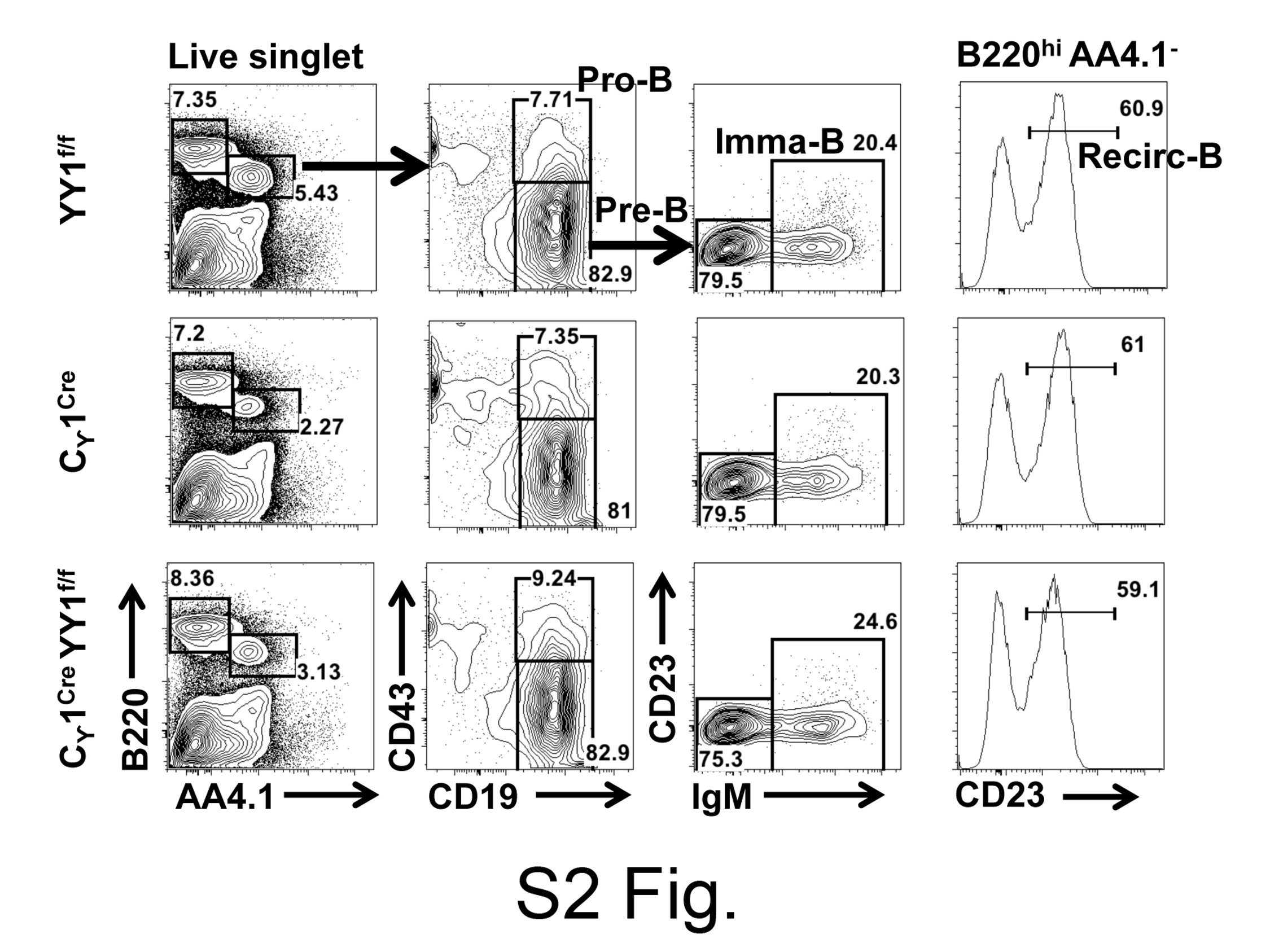

Supplement: S2 Fig — (A) Bone marrow cells from yy1f/f, γ1CRE, and yy1f/f γ1CRE mice were stained with various antibodies to identify B cell developmental subsets, and re-circulating mature B cells. After doublet and dead cell discrimination, progenitor B cells (Pro-B) were phenotyped as B220+AA4.1+CD19+CD43+ cells; precursor B cells (Pre-B) were phenotyped as B220+AA4.1+CD19+CD43-CD23-IgM- cells; immature B cells (Imma-B) were phenotyped as B220+AA4.1+CD19+CD43-CD23+/-IgM+ cells and recirculating mature B cells (Recirc-B) were phenotyped as B220+AA4.1-CD23+ cells. (TIF) [file pone.0155311.s002.tif]

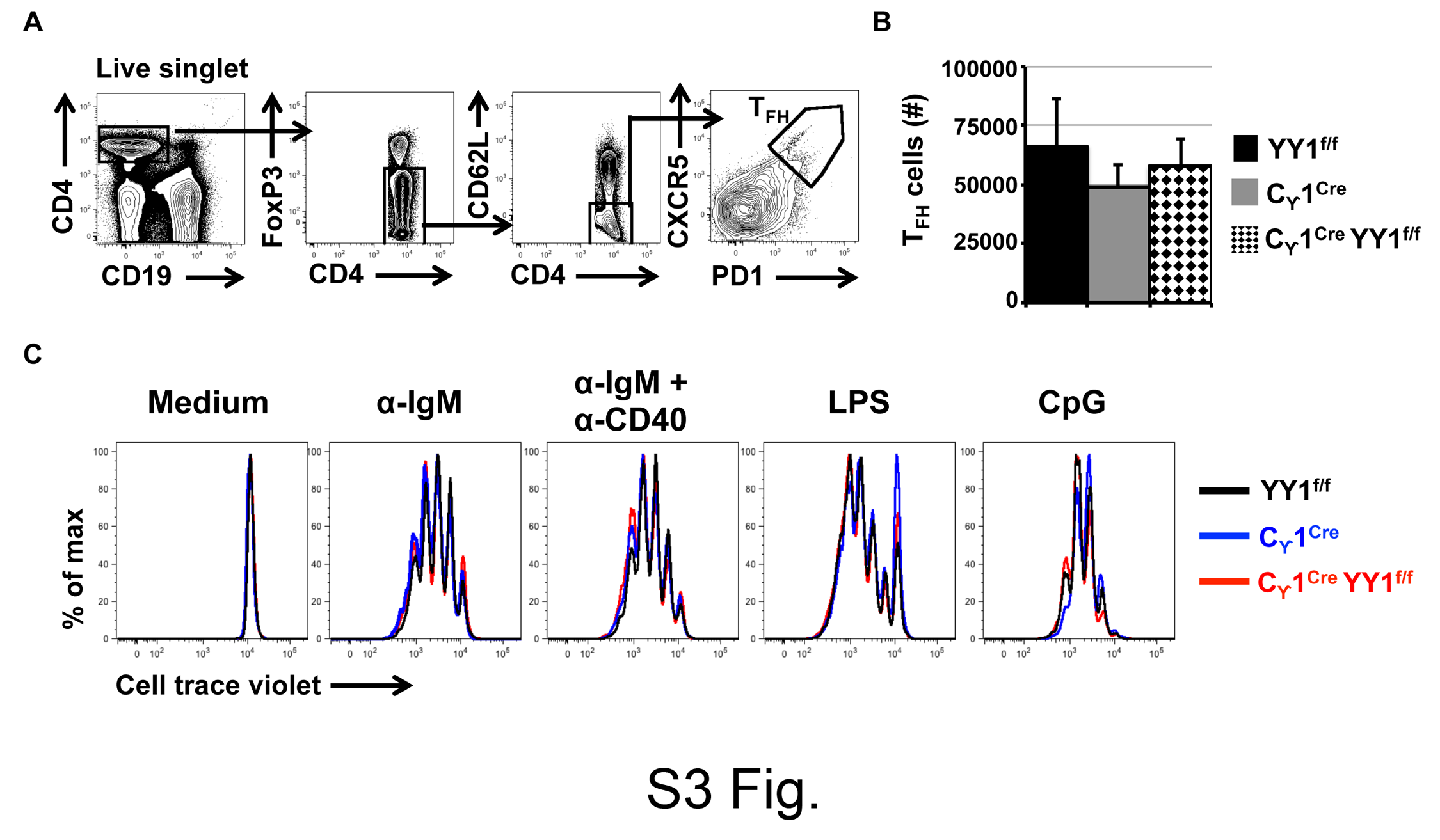

Supplement: S3 Fig — (A) Representative staining of TFH cells in YY1f/f mice. Live singlet cells were gated and subsetted into TFH (CD4+FoxP3−CD62L-PD1hiCXCR5hi) cells. (B) Number of TFH cells per spleen of non-immunized YY1f/f, Cγ1Cre and Cγ1Cre YY1f/f mice. Representative gating strategy to identify TFH (CD4+FoxP3−CD62L-PD1hiCXCR5hi) cells as shown in A (left). (C) MACS-sorted CD23+ Follicular B cells from YY1f/f, Cγ1Cre and Cγ1Cre YY1f/f mice were labeled with CFSE and stimulated for 60 hours with anti-IgM (20 μg/ml), anti-IgM + anti-CD40 (2.5 μg/ml), LPS (5 μg/ml), or CpG (1 μM). At the end of the culture, live and dead cells were identified by TO-PRO-3 staining. CFSE dilution in the live cells is shown in the figure. Representative results are from three independent experiments. (TIF) [file pone.0155311.s003.tif]
